# Interpretable machine learning models for predicting venous thromboembolism in the intensive care unit: an analysis based on data from 207 centers

**DOI:** 10.1186/s13054-023-04683-4

**Published:** 2023-10-24

**Authors:** Chengfu Guan, Fuxin Ma, Sijie Chang, Jinhua Zhang

**Affiliations:** Department of Pharmacy, Fujian Maternity and Child Health Hospital College of Clinical Medicine for Obstetrics and Gynecology and Pediatrics, Fujian Medical University, #18 Daoshan Road, Fuzhou, 350001 China

**Keywords:** Machine learning, Venous thromboembolism, Critically ill, Prediction model

## Abstract

**Background:**

Venous thromboembolism (VTE) is a severe complication in critically ill patients, often resulting in death and long-term disability and is one of the major contributors to the global burden of disease. This study aimed to construct an interpretable machine learning (ML) model for predicting VTE in critically ill patients based on clinical features and laboratory indicators.

**Methods:**

Data for this study were extracted from the eICU Collaborative Research Database (version 2.0). A stepwise logistic regression model was used to select the predictors that were eventually included in the model. The random forest, extreme gradient boosting (XGBoost) and support vector machine algorithms were used to construct the model using fivefold cross-validation. The area under curve (AUC), accuracy, no information rate, balanced accuracy, kappa, sensitivity, specificity, precision, and *F*1 score were used to assess the model's performance. In addition, the DALEX package was used to improve the interpretability of the final model.

**Results:**

This study ultimately included 109,044 patients, of which 1647 (1.5%) had VTE during ICU hospitalization. Among the three models, the Random Forest model (AUC: 0.9378; Accuracy: 0.9958; Kappa: 0.8371; Precision: 0.9095; *F*1 score: 0.8393; Sensitivity: 0.7791; Specificity: 0.9989) performed the best.

**Conclusion:**

ML models can be a reliable tool for predicting VTE in critically ill patients. Among all the models we had constructed, the random forest model was the most effective model that helps the user identify patients at high risk of VTE early so that early intervention can be implemented to reduce the burden of VTE on the patients.

**Supplementary Information:**

The online version contains supplementary material available at 10.1186/s13054-023-04683-4.

## Introduction

Venous thromboembolism (VTE), which includes deep vein thrombosis (DVT) and pulmonary embolism (PE), is a chronic disease that frequently recurs. About 30% of patients with VTE are estimated to recur within ten years [1, 2]. VTE often leads to patient death, long-term disability, and bleeding associated with anticoagulation therapy and is one of the major contributors to the global burden of disease [3]. Although PE-related mortality has decreased yearly, nearly 10% of PE patients die within 30 days of diagnosis [4]. In addition, VTE carries a significant economic burden. The US healthcare system spends $7–10 billion annually related to VTE events, and Europe spends €1.5–3.3 billion [5, 6]. Critically ill patients are at much greater risk of VTE than medically hospitalized patients. Critically ill patients face general risk factors for VTE, including factors like age, obesity, a prior history of VTE, and cancer. Moreover, they are also susceptible to ICU-specific risk factors such as immobilization, the use of central venous catheters (CVC), and mechanical ventilation [7–9]. Although anticoagulants are clinically given to critically ill patients to prevent thrombosis, the incidence of VTE in critically ill patients is still high [10]. Therefore, identifying patients at high risk of VTE through risk assessment models can help in early prevention and timely treatment.

Machine Learning (ML) is the discipline in which computers use algorithms to learn from data and can recognize underlying patterns in the data. ML has powerful computational and data-fitting capabilities to find complex relationships between large amounts of data. These features make ML well-suited for complex clinical datasets, and its use in clinical research is increasing yearly [11]. In previous studies, the ML model demonstrated excellent performance [12, 13]. While the performance of ML models is excellent, the black-box (i.e., data goes in, decisions come out, and inputs to outputs are opaque) nature of ML similarly limits its application [14, 15]. Therefore, understanding why and how models make decisions is critical to using models in clinical practice. Algorithms for interpreting ML models have recently emerged, and these algorithms can increase users' understanding and trust in ML models [16].

In this paper, we report the development of an ML model for predicting VTE in critically ill patients. We also used an interpretable algorithm for the ML model to interpret the predictions of the model.

## Methods

### Data source and population

Data for this study were extracted from the eICU Collaborative Research Database (version 2.0) [17]. The database is a multicenter, publicly available ICU database containing de-identified, high-granularity medical data on 200,859 ICU admissions from 208 centers across the United States from 2014 to 2015 [18]. The eICU Collaborative Research Database included vital signs, care plan documentation, disease severity measures, diagnoses, treatments, and laboratory results recorded by care providers during a patient's ICU stay. This study's data extractor and processor was granted a license to use the data (certification number: 11678655). Informed consent was waived due to the de-identified nature of the data.

**Fig. 1:**
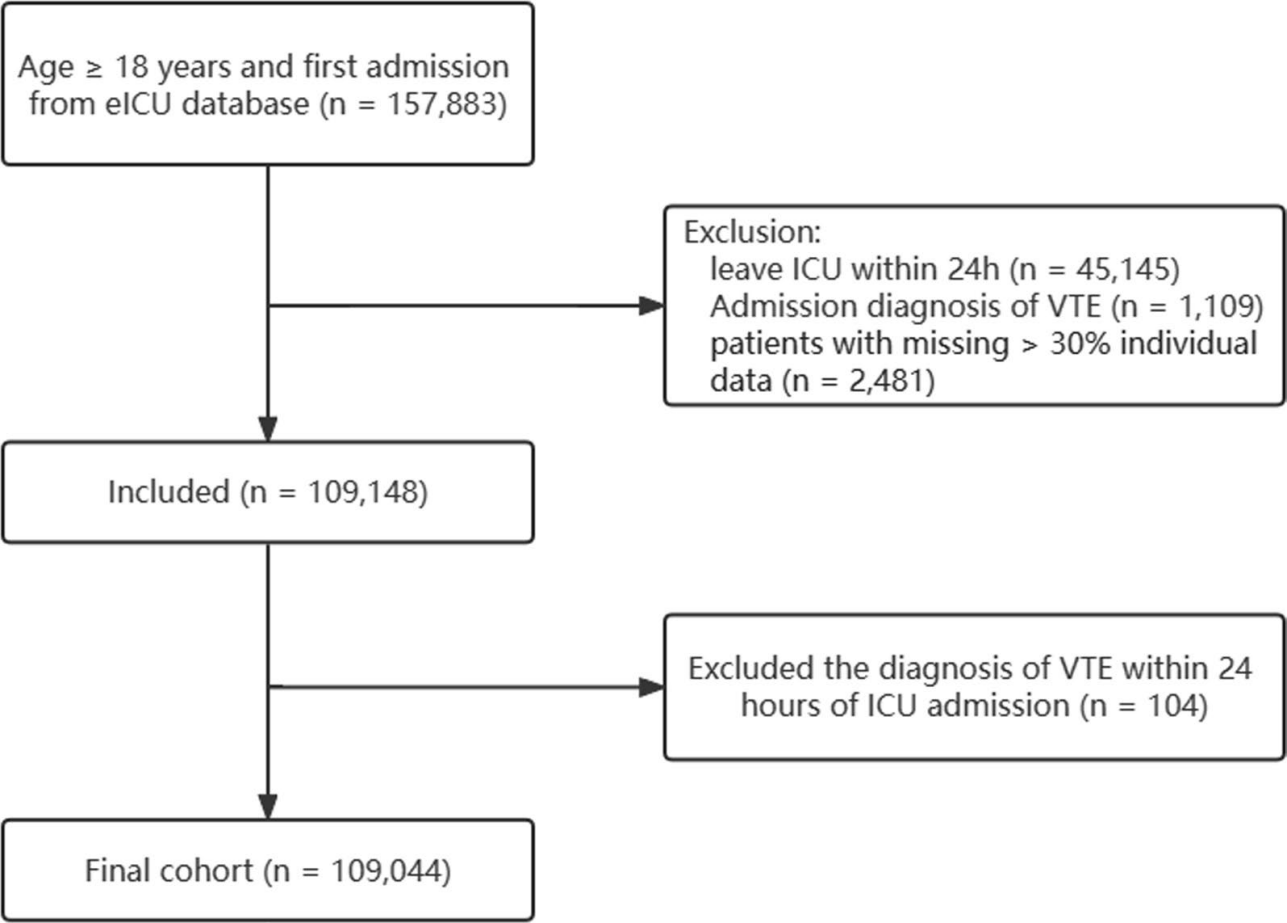
Flowchart of patient selection. Abbreviations: VTE, venous thromboembolism; ICU, intensive care unit

In this study, all patients aged greater than or equal to 18 years we considered for inclusion, and for patients with multiple ICU admissions, only the first admission was considered. Exclusion criteria were as follows:(1) ICU stay of less than 24 h; (2) VTE as an admission diagnosis; (3) diagnosis of VTE within 24 h of ICU admission; and (4) individual data missing greater than 30%. The flowchart for study cohort selection is shown in Fig. 1.

### Feature extraction

Baseline information was extracted using Structured Query Language (SQL) for the 24 h following the patient's admission to the ICU. Demographic information included age, gender, body mass index (BMI), Acute Physiology and Chronic Health Evaluation IV score (APACHE IV score), previous history of VTE, history of cancer, and Glasgow Coma Scale (GCS). Laboratory parameters included hematocrit, hemoglobin, platelet count, white blood cell count, albumin, blood urea nitrogen (BUN), serum creatinine, international normalized ratio (INR), prothrombin time (PT), partial thromboplastin time (PTT), total bilirubin, alanine aminotransferase (ALT), and aspartate transaminase (AST). The treatment received by the patient on the first day of admission to the ICU included mechanical ventilation, CVC, vasopressin, sedative, transfusion of fresh frozen plasma, platelet transfusion, transfusion of packed red blood cells, pharmacologic prophylaxis, and graduated compression stockings. The principal diagnosis on admission included cardiovascular condition, respiratory condition, gastrointestinal condition, renal condition, neurologic condition, metabolic condition, trauma, and other conditions. Diseases that patients suffer from included cancer, respiratory failure, heart failure, end-stage renal disease (ESRD), and sepsis. We selected the maximum value for variables measured multiple times in 24 h. We used multiple interpolation to interpolate missing values [19]. Multiple interpolation generated multiple complete datasets by fitting the possible values of the missing data multiple times through the model. Afterwards, multiple interpolation analyzed the generated datasets and combined the results of multiple analyses to finally obtain a comprehensive estimate and statistical inference. In contrast to single interpolation, multiple interpolation filled in missing values multiple times, which quantified the uncertainty in estimating missing values and avoided generating incorrect accuracies [20]. Details on missing values were available in Additional file 1: Fig. S1.

### Outcomes

The primary outcome of this study was new VTE during ICU hospitalization, including DVT, PE, or both.

### Statistical analysis

Depending on whether or not it conformed to a normal distribution, continuous variables were presented as mean (standard deviation) or median (quartiles 1–3). Categorical variables were described as frequencies (percentages). We compared the clinical characteristics of the VTE and non-VTE groups using the Student t-test for normally distributed continuous variables and the Mann–Whitney *U* test for non-normally distributed ones. Differences in categorical variables were compared using the *χ*2 test or Fisher's precision probability test. A two-sided *P* value < 0.05 was regarded as statistically significant. A stepwise logistic regression model was used to select the predictors that were ultimately included in the model. Akaike Information Criterion (AIC) was used as a selection criterion for stepwise feature selection [21]. We calculated the AIC at each step while using forward selection and backward elimination of predictor variables, stopping when further addition or removal of variables no longer improved the AIC, thus obtaining the model with the lowest AIC.

In addition, we used the DALEX package to improve the interpretability of the final model [16]. The DALEX package contains various explainers that help understand the relationship between input variables and model outputs. The DALEX package allows us to understand the importance of the variables in the model, the relationship between the variables and the clinical outcomes, and assess each variable's contribution to individual predictions.

### Study design

The eICU Collaborative Research Database used in this study is a multicenter database of 208 hospitals. We used hospitals as the basic unit and randomly selected hospitals containing about 70% of the patients in the final cohort as the training set and the remaining hospitals containing about 30% as the validation set for external validation of the model. We described the hospital IDs included in the training and validation sets in Additional file 1: Table S1 and described the demographic and clinical characteristics of the training and validation sets in Additional file 1: Table S2. Since our data was characterized by class imbalance, high dimensionality and large sample size, we selected from common machine learning algorithms that are more suitable for our data. We finally chose random forest, extreme gradient boosting (XGBoost), and support vector machine (SVM) algorithms for model construction and tuned the hyperparameters using a randomized search strategy with five-fold cross-validation. Five-fold cross-validation means dividing the dataset into five mutually exclusive subsets, each acting as a fold. Four folds were used in each round as the training set, leaving one fold as the test set. Repeat this process five times, ensuring each fold has acted as a test set. Cross-validation reduces model overfitting and improves robustness. For imbalanced data, machine learning models may tend to favor the dominant class while neglecting the minority class. To address data imbalance, we adjusted the classification threshold. Typically, the model's default classification threshold is set at 0.5, meaning that a sample is classified as the positive class when the model's output probability is greater than 0.5 and as the negative class otherwise. However, in the case of class imbalance, this default threshold may not be the optimal choice. After considering various model performance metrics, we ultimately selected a threshold of 0.2. This means that when the model's output probability is greater than 0.2, it predicts a positive result; otherwise, it predicts a negative result. The area under curve (AUC) of the receiver operating characteristic (ROC) curve, accuracy, no information rate, balanced accuracy, kappa, sensitivity, specificity, precision, and F1 scores were used to assess the performance of the models. This study's statistical analysis and model construction were based on R version 4.3.0.

## Results

### Baseline characteristics

A total of 109,044 patients were enrolled in the cohort of this study, with 72,742 patients in the training set and 36,302 patients in the validation set. We divided the patients into VTE and non-VTE groups based on whether VTE occurred during ICU hospitalization, with 1647 (1.5%) patients in the VTE group and 107,397 (98.5%) patients in the non-VTE group. Baseline differences between the VTE and non-VTE groups were shown in Table 1. Patients who developed VTE during their ICU stay had a higher BMI than the non-VTE group. Previous history of VTE and history of cancer were higher in the VTE group than in the non-VTE group. In the VTE group, ICU admissions for respiratory disease and sepsis were higher than in the non-VTE group. Compared with the non-VTE group, the VTE group had higher prevalence of cancer, respiratory failure, heart failure, and sepsis; and higher rates of mechanical ventilation, CVC, use of vasopressors, and transfusion of fresh frozen plasma and packed red blood cells. The maximum values of platelet count, white blood cell count, BUN, serum creatinine, total bilirubin, ALT, and AST were higher in the VTE group than in the non-VTE group. In addition, the proportion of pharmacologic prevention of VTE was slightly higher in the VTE group than in the non-VTE group. In contrast, the proportion of mechanical prevention was not significantly different between the two groups.

**Table 1:** Demographic and clinical characteristics between VTE and non-VTE group

Characteristics	With VTE (*n* = 1647)	Without VTE (*n* = 107,397)	*p*
Demographics			
Age	67.0 (55.0–76.0)	66.0 (54.0–76.0)	0.569
Male, *n* (%)	882 (53.6)	58,243 (54.2)	0.583
Body mass index, kg/m^2^	29.1 (24.4–35.2)	27.5 (23.5–32.9)	< 0.001
APACHE IV score	58.0 (43.0–80.0)	54.0 (40.0–71.0)	< 0.001
Past history of VTE, *n* (%)	457 (27.7)	4641 (4.3)	< 0.001
History of cancer, *n* (%)	346 (21.0)	15,722 (14.6)	< 0.001
Glasgow coma scale	14.0 (9.0–15.0)	14.0 (10.0–15.0)	0.311
Principal diagnosis on admission, n (%)			
Cardiovascular condition	347 (21.1)	29,364 (27.3)	< 0.001
Respiratory condition	576 (35.0)	17,329 (16.1)	< 0.001
Gastrointestinal condition	135 (8.2)	11,053 (10.3)	0.005
Renal condition	22 (1.3)	1847 (1.7)	0.233
Neurologic condition	155 (9.4)	19,485 (18.1)	< 0.001
Sepsis	289 (17.5)	14,925 (13.9)	< 0.001
Metabolic condition	28 (1.7)	4368 (4.1)	< 0.001
Trauma	44 (2.7)	4901 (4.6)	< 0.001
Other condition	51 (3.1)	4125 (3.8)	0.118
Diagnosed diseases, *n* (%)			
Cancer	220 (13.4)	4792 (4.5)	< 0.001
Respiratory failure	759 (46.1)	26,110 (24.3)	< 0.001
Heart failure	247 (15.0)	10,064 (9.4)	< 0.001
End stage renal disease	56 (3.4)	3118 (2.9)	0.234
Sepsis	444 (27.0)	15,543 (14.5)	< 0.001
Treatments, *n* (%)			
Mechanical ventilation	688 (41.8)	40,687 (37.9)	0.001
Central venous catheter	298 (18.1)	15,091 (14.1)	< 0.001
Vasopressor	422 (25.6)	20,636 (19.2)	< 0.001
Sedative	630 (38.3)	40,071 (37.3)	0.434
Transfusion of blood product			
Platelet	42 (2.6)	2301 (2.1)	0.258
Fresh frozen plasma	102 (6.2)	3935 (3.7)	< 0.001
Packed red blood cells	199 (12.1)	10,250 (9.5)	0.001
Laboratory test results			
Hematocrit, %	36.5 (31.4–41.3)	37.4 (32.5–42.0)	< 0.001
Hemoglobin, g/dl	11.9 (10.1–13.8)	12.3 (10.6–14.0)	< 0.001
Platelet, K/uL	226.0 (166.0–303.0)	221.0 (169.0–286.0)	0.007
While blood cells, K/uL	12.9 (9.2–18.4)	12.2 (8.9–16.9)	< 0.001
Albumin, g/dL	3.0 (2.6–3.6)	3.4 (2.8–3.8)	< 0.001
Blood urea nitrogen, mg/dL	25.0 (16.0–40.0)	22.0 (15.0–36.0)	< 0.001
Creatinine, mg/dL	1.2 (0.9–2.0)	1.1 (0.8–1.8)	< 0.001
INR	1.2 (1.1–1.5)	1.4 (1.1–1.9)	< 0.001
PT, s	13.3 (11.5–16.2)	15.9 (13.5–21.4)	< 0.001
PTT, s	31.9 (27.5–39.5)	35.1 (29.4–42.6)	< 0.001
Total bilirubin, mg/dL	0.7 (0.5–1.1)	0.6 (0.4–1.0)	< 0.001
ALT, U/L	29.0 (18.0–56.0)	26.0 (17.0–45.0)	< 0.001
AST, U/L	32.0 (20.0–72.0)	30.0 (20.0–58.0)	< 0.001
VTE prophylaxis method, *n* (%)			
Pharmacologic prophylaxis	881 (53.5)	54,782 (51.0)	0.046
Graduated compression stockings	743 (45.1)	50,133 (46.7)	0.206

*VTE* venous thromboembolism, *BMI* body mass index, *GCS* glasgow coma scale, *ESRD* end stage renal disease, *CVC* central venous catheter, *Bun* blood urea nitrogen, *INR* international standard ratio, *PT* prothrombin time, *PTT* partial thromboplastin time, *ALT* alanine aminotransferase, *AST* aspartate transaminase

### Feature selection and model performance comparisons

We collected a total of 43 clinical and biological variables within 24 h of the patient's ICU admission. Through stepwise logistic regression, we finally selected 24 variables, which were age, gender, BMI, previous history of VTE, history of cancer, cancer, respiratory failure, heart failure, sepsis, hematocrit, hemoglobin, platelet count, white blood cell count, albumin, serum creatinine, INR, PT, PTT, total bilirubin, ALT, AST, transfusion of packed red blood cells, mechanical ventilation, and CVC.

**Fig. 2:**
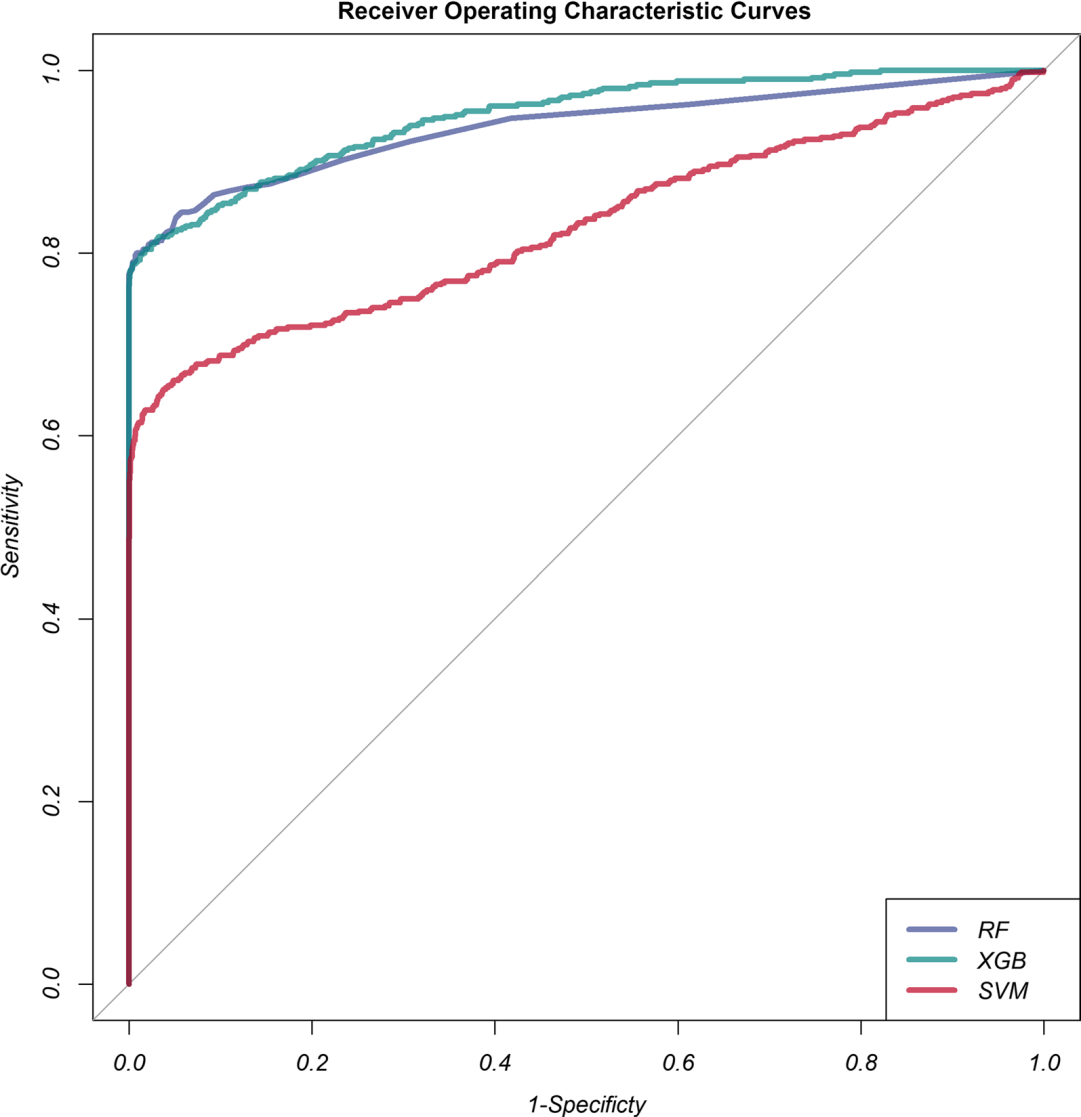
Receiver operating characteristic curves of the three models for predicting VTE. RF, random forest; XGB, eXtreme gradient boosting; SVM, support vector machine

Random forest, XGBoost and SVM algorithms were used to construct models. The fivefold cross-validated random search strategy resulted in the finalization of the hyperparameters for fandom forest as mtry = 12; for XGBoost as nrounds = 46, lambda = 0.0002833363, alpha = 0.1278563 and eta = 0.3265631; and SVM as sigma = 0.03212153 and *C* = 0.1837905. We used AUC, accuracy, no information rate, balanced accuracy, kappa, sensitivity, specificity, precision, and F1 scores to comprehensively evaluate the model's performance. XGBoost had the largest AUC (0.9492) and sensitivity (0.7810), followed by random forest (AUC: 0.9378; sensitivity: 0.7791) and SVM (AUC: 0.8290; sensitivity: 0.5911) (Table 2). Figure 2 described the ROC curves for the three models. The accuracy, kappa, specificity, precision and F1 scores of random forest were higher than those of XGBoost and SVM, as shown in Table 1. Compared to the random forest, XGBoost had a higher sensitivity, i.e., the number of underreporting (false negatives) was slightly lower in the XGBoost model than in the random forest model. However, the precision of the random forest was higher than XGBoost, i.e., the number of false positives (false positives) was lower in the random forest model than in the XGBoost model. The random forest had better clinical utility compared to XGBoost and SVM.

**Table 2:** Performance of three machine learning models for predicting VTE in critically ill patients

Models	AUC	Accuracy	No Information Rate	Balanced Accuracy	Kappa	Precision	F1 score	Sensitivity	Specificity
RF	0.9378	0.9958	0.9858	0.8890	0.8371	0.9095	0.8393	0.7791	0.9989
XGBoost	0.9492	0.9947	0.9858	0.8894	0.8041	0.8344	0.8068	0.7810	0.9978
SVM	0.8290	0.9899	0.9858	0.7934	0.6186	0.6602	0.6237	0.5911	0.9956

*AUC* area under curve, *RF* random forest, *XGBoost* eXtreme gradient boosting, *SVM* support vector machine

### Explainability

**Fig. 3:**
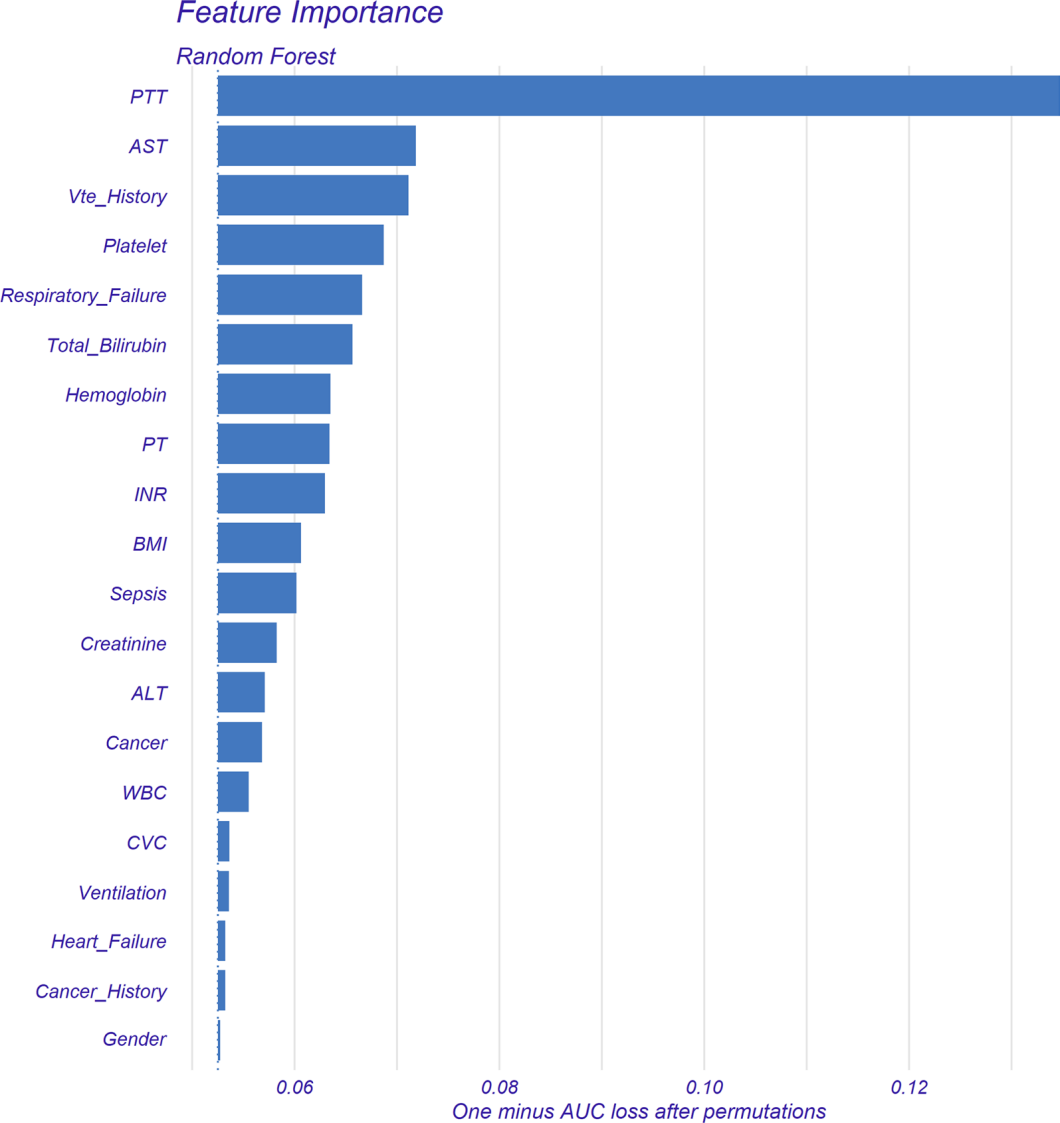
Feature importance derived from random forest model. This figure is the result of the DALEX package. The X-axis represents the loss in AUC calculated after randomly permuting the feature compared to the original AUC. The greater this loss, the higher the model's importance of this feature. Abbreviations: PTT, partial thromboplastin time; AST, aspartate transaminase; PT, prothrombin time; INR, international standard ratio; BMI, body mass index; ALT, alanine aminotransferase; WBC, white blood cell; CVC, central venous catheter

We calculated feature importance using the DALEX package and showed the top 20 clinical variables in terms of importance in Fig. 3. In Additional file 1: Figs. S2–6, we also described the effect (positive or negative) of clinical characteristics on the model. Characteristics associated with increased incidence of VTE were higher age, BMI, platelet count, white blood cell count, serum creatinine, ALT, AST, and total bilirubin. And lower PTT, PT, and INR were associated with an increased incidence of VTE. In addition, a history of prior VTE, a diagnosis of cancers, heart failure, respiratory failure, sepsis, and treatment with CVC, mechanical ventilation, and transfusion of packed red blood cells were also helpful in predicting VTE. Gender and cancer history were not strongly associated with VTE prediction. We also found that albumin, hematocrit, and hemoglobin were associated with an increased risk of VTE in a U-shaped curve. We named the final model Alfalfa-ICU-VTE (“Alfalfa” is the name of our team, representing happiness and luck).

## Discussion

In this study, based on 24 variables collected within 24 h of ICU admission, we developed three ML models to provide individual predictions of whether VTE occurs in critically ill patients during their ICU stay. The random forest model demonstrated the best performance. Through feature importance analysis, we identified the 20 clinical variables that had the greatest impact on the prediction of VTE, in descending order of importance: PTT, AST, history of previous VTE, platelet count, respiratory failure, total bilirubin, hemoglobin, PT, INR, BMI, sepsis, serum creatinine, ALT, cancer, white blood cell count, CVC, mechanical ventilation, heart failure, history of cancer, and gender. In addition, we described how these variables affected the random forest model. Finally, through the interpretable algorithm of the ML model, we learned how the model obtained individual case predictions.

In many previous studies, ML models have shown excellent performance, but these models suffer from a lack of interpretability, i.e., these models were black boxes. Users can input data to obtain outputs, and it was unclear how the model generates predictions, which limited the use of ML models in clinical settings. Even if the model has demonstrated a high degree of accuracy, the lack of understanding of why and how the model makes predictions inevitably causes concerns when clinicians want to treat or prevent patients based on the model's predictions. Similarly, patient cooperation will be poor if the physician doesn't understand why the algorithm is making predictions. Especially in complex cases with significant healthcare consequences, the black-box nature of ML models will greatly hinder their application. The 2018 European General Data Protection Regulation stated that when using ML algorithms for decision-making, individuals have the right to obtain meaningful information about the logic involved as well as the implications and expected consequences of such processing. The regulation conveyed concerns about the opaque predictions of ML models [22, 23]. The interpretable ML models we built help users better understand the decision-making process of the models, thus making them more reliable and transparent. Our model also provided insights into the contribution of predictor variables to individual predicted outcomes, aiding caregivers in the development of more flexible care plans tailored to specific patient conditions. Furthermore, our model effectively identified patients at high risk of thrombosis, allowing for the prioritization of limited healthcare resources towards those requiring special attention, thereby optimizing resource allocation. Simultaneously, this approach assisted in alleviating the financial burden on patients, particularly those in less favorable financial situations. By strengthening the monitoring of high-risk thrombosis patients, it was conducive to early detection and treatment of thrombosis, reducing its impact on patients.

**Fig. 4:**
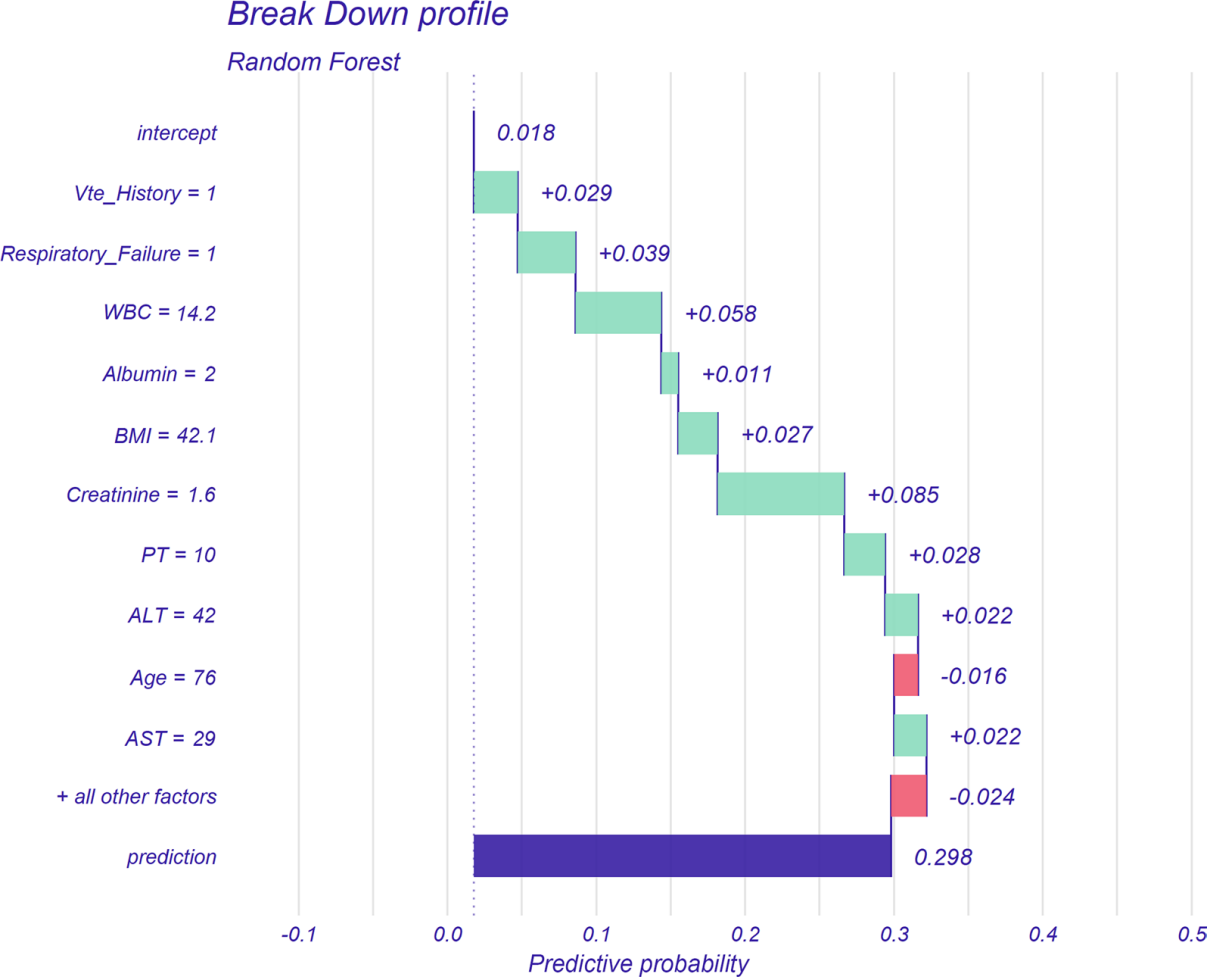
Explaining of patient prediction results. This figure was made with the DALEX package for explaining random forest model predictions. Abbreviations: WBC, while blood cells; BMI, body mass index; PT, prothrombin time; ALT, alanine aminotransferase; AST, aspartate transaminase

To further explore the contribution of these clinical features to individual patient predictive outcomes, we randomly selected four patients from the validation cohort for presentation. With interpretable algorithms, we can visualize which clinical indicators in a given patient increased the prediction of VTE and which variables decreased the prediction. We showed one of these patients in the main text, and the remaining three are available in the supplemental material (Additional file 1: Figs. S7–9). This patient was a 76-year-old male with a BMI of 42.1. He had a history of previous VTE but no history of cancer, and he presented with respiratory failure. Laboratory markers on the first day of ICU admission showed a hematocrit of 43.3%, hemoglobin level of 15.4 g/dl, platelet count of 115 K/uL, white blood cell count of 14.2 K/uL, albumin level of 2 g/dL. His serum creatinine was 1.6 mg/dL, INR was 0.8, PT was 10 s, PTT was 29.7 s, total bilirubin was 0.7 mg/dL, ALT was 42 U/L, and AST was 29 U/L. He received mechanical ventilation treatment and did not require a transfusion of packed red blood cells or CVC (Fig. 4). The ML model predicted a 29.8% risk of VTE based on the patient's clinical characteristics within 24 h of admission to the ICU, with serum creatinine, hemoglobin, comorbid respiratory failure, history of previous VTE, and PT being the top five contributors to the increased risk of VTE, whereas age reduced the model's prediction of VTE. The predicted outcome of the ML model was that the patient had a VTE, and the actual outcome was that the patient had a VTE while in the ICU (true positive).

Our findings indicated that lower PTT, PT and INR were associated with an increased risk of VTE in critically ill patients. PTT was a blood test that characterizes blood coagulation and was related to the intrinsic and common pathways of coagulation. Several population-specific studies have also shown that low levels of PTT were associated with an increased risk of VTE [24, 25]. Lower PTT may be due to increased coagulation factor activity in the intrinsic or common pathway or resistance to activated protein C, increasing the risk of thrombosis [24, 25]. PT was another coagulation test to assess tissue factors and common coagulation pathways. Lower levels of PT were associated with an increased risk of VTE, possibly due to increased activity of coagulation factors II, V, VII, X and fibrinogen [26]. INR was a mathematical conversion form of PT and was related to VTE similarly to PT.

Our findings showed that higher ALT, AST and total bilirubin were associated with an increased risk of VTE. In some previous studies, researchers observed that abnormal liver function may increase the incidence of thrombosis in patients, which was similar to our findings [27–30]. Coagulation factor VIII is one of the most potent drivers of thrombin generation, and the increased risk of thrombosis in patients with abnormal hepatic function may be associated with significantly elevated plasma levels of coagulation factor VIII [31]. In patients with hepatic insufficiency, high levels of von Willebrand factor and underexpressed low-density lipoprotein receptor-associated protein together maintain high plasma levels of factor VIII [32, 33]. Von Willebrand factor binds to factor VIII and protects it from cleavage and premature clearance by plasma proteases [34]. Low-density lipoprotein receptor-associated protein mediates cellular uptake and degradation of factor VIII [35].

Like a previous study that prospectively explored risk factors for VTE in ICU patients, our results showed that critically ill patients with a history of VTE were at higher risk for VTE, reaffirming that VTE was a relapsing disease [7]. In addition, we found that critically ill patients with comorbid cancers were more likely to develop VTE. Cancer patients are often in a hypercoagulable state. The presence of cancer tends to activate the coagulation cascade, promote platelet activation, and increase the aggregation status of blood cells, such as platelets and leukocytes [36]. In addition, cancer treatments such as chemotherapy and targeted therapies may promote thrombosis through mechanisms that are not fully understood [37, 38]. The findings also pointed to sepsis as similarly increasing the risk of VTE. Sepsis is a syndrome of the systemic inflammatory response caused by infection, and inflammation is considered a common pathway for VTE formation triggered by many risk factors. Inflammation of the vessel wall induces thrombosis, and the inflammatory and coagulation systems are coupled through common activation pathways [39]. The systemic inflammatory response induced by sepsis leads to activation and depletion of coagulation factors and platelets, impaired fibrinolytic function, disruption of the vascular endothelial barrier, and loss of physiologic antithrombotic factors such as thrombomodulin [40]. Our findings also showed that respiratory and heart failure were risk factors for VTE, which was similar to the results of previous studies [41, 42].

We also found that receiving CVC and mechanical ventilation increased the risk of VTE. CVC and mechanical ventilation are frequently used in the ICU as important therapeutic measures to maintain vital signs in critically ill patients. Still, their presence also puts critically ill patients at increased risk of thrombosis. When CVC is exposed to the bloodstream due to the lack of a normal endothelial layer of the blood vessel wall, CVC cannot inhibit platelet adhesion and coagulation. Therefore, in some cases, CVC activates the contact pathway, ultimately leading to thrombosis [43]. Decreased venous return and restricted mobility due to increased intrathoracic pressure in patients undergoing mechanical ventilation may be responsible for the increased risk of thrombosis [9]. In an accompanying clinical trial, researchers found that mechanical ventilation led to pulmonary and systemic coagulation disorders in patients, which may be another reason mechanical ventilation increases the risk of thrombosis [44]. A previous retrospective cohort study also suggested that mechanical ventilation is an independent risk factor for VTE [45]. Furthermore, it should be noted that mechanically ventilated patients often require lung scans, which may increase the rate of PE diagnosis.

This study has some strengths and weaknesses. We used advanced ML techniques for modeling. The powerful computational and fitting capabilities of ML algorithms enable the construction of complex models. In addition, we used the DALEX package to explain the decision-making process of the ML model, helping clinical users better understand the model's predictive process. Moreover, our study included 109,044 patients from 207 centers, giving our model some generalizability. The limitation of this study was that it was retrospective, and inevitably there will be some bias. Second, the study lacked validation in prospective clinical trials to determine the exact performance of the model in the real world. Thirdly, the interpolation values generated by the multiple interpolation method were based on the estimation of the statistical model, and thus there was an estimation error. This meant that the interpolated values may have some deviation from the true values, which may have some impact on the performance of the machine learning model. Finally, immobilization is one of the important risk factors for VTE, yet this clinical variable was not included in our model. Although information on immobilization after ICU admission was available in the eICU database, it was not available before ICU admission. We planned to use the clinical characteristics of patients within 24 h of ICU admission for prediction and therefore did not include immobilization in the predictor variables. Subsequently, we will integrate the model into a web page to make it easy to use as an online tool. Additionally, we have devised plans to integrate the model with the hospital's case management system, automating the assessment of patients' VTE risk. We will then embark on a prospective study within our hospital to validate the model's performance in real-world scenarios. Depending on the model's performance within our single-center setting, we will contemplate its extension for prospective multicenter validation.

## Conclusion

ML modeling can be a reliable tool for predicting VTE in critically ill patients. Among all the models we have constructed, the random forest model was the most effective model that helped the user identify patients at high risk of VTE early so that early intervention can be implemented to reduce the burden of VTE on the patients.

### Supplementary Information

**Additional file 1.** Supplementary Appendix.

## Data Availability

The datasets presented in the current study are available in the eICU Collaborative Research Database (version 2.0) (https://physionet.org/content/eicu-crd/2.0/). Though datasets are de-identifed, restrictions have been imposed on data sharing since they contain sensitive information. Before accessing the data, the researcher must sign the relevant convention. To access the data, interested researchers must meet all of the following requirements: be a credentialed user of https://physionet.org/, finish required training and sign the data use agreement for the project. All the code used for this project is available on github (https://github.com/bbpob/alfalfa-vte).
