# Supplementary material for: Interpretable machine learning models for predicting venous thromboembolism in the intensive care unit: an analysis based on data from 207 centers
Source: Crit Care. 2023 Oct 24;27:406. doi: 10.1186/s13054-023-04683-4 (PMC10598960; doi:10.1186/s13054-023-04683-4)
Supplement: Supplementary file 1 — Additional file 1. Supplementary Appendix. [file 13054_2023_4683_MOESM1_ESM.docx]

**Supplemental Content**

**Table S1:** Hospital id included in the training set and validation set.

**Table S2:** Demographic and clinical characteristics between training set and validation set.

**Figure S1:** Percentage of missing variables.

**Figure S2:** Relationship between age, albumin, hematocrit, hemoglobin and predicted outcomes.

**Figure S3:** Relationship between BMI, INR, platelet, PT, PTT, WBC and predicted outcomes.

**Figure S4:** Relationship between ALT, AST, creatinine, total bilirubin and predicted outcomes.

**Figure S5:** Relationship between cancer, cancer history, gender, heart failure, respiratory failture, vte history and predicted outcomes.

**Figure S6:** Relationship between CVC, RBW input, sepsis, mechanical ventilation and predicted outcomes.

**Figure S7:** Explaining of patient prediction results.

**Figure S8:** Explaining of patient prediction results.

**Figure S9:** Explaining of patient prediction results.

**Table S1. Hospital id included in the training set and validation set.**

| Dataset | Hospital IDs |
| --- | --- |
| Training set | 56,68,69,84,85,86,90,91,93,94,95,96,102,112,115,120,125,133,135,136,138,151,155,156,158,164,174,175,179,180,196,199,201,202,205,206,207,208,210,215,217,220,224,226,227,243,244,245,246,248,249,250,251,252,253,254,256,258,259,264,266,267,268,269,271,272,273,275,277,279,280,281,282,283,300,301,307,312,318,331,336,337,338,345,350,353,357,358,360,364,365,382,383,384,387,389,390,391,392,394,396,397,398,399,402,403,404,405,407,408,411,413,416,417,419,420,421,423,424,428,429,433,434,435,436,439,440,443,444,445,452,458,459 |
| Validation set | 58,59,60,61,63,66,67,71,73,79,83,92,108,110,122,123,131,140,141,142,143,144,146,148,152,154,157,165,167,171,176,181,182,183,184,188,194,195,197,198,200,203,204,209,212,262,263,265,303,310,323,328,342,351,352,355,356,363,381,385,386,388,393,400,401,409,412,414,422,425,437,438,447,449 |

**Table S2. Demographic and clinical characteristics between training set and validation set.**

| Characteristics | Training set (n = 72,742) | Validation set (n = 36,302) | p |
| --- | --- | --- | --- |
| VTE | 1131 (1.6) | 516 (1.4) | 0.089 |
| Demographics |  |  |  |
| Age | 65.0 (53.0-76.0) | 66.0 (54.0-77.0) | <0.001 |
| Male, n (%) | 33,303 (45.8) | 16,616 (45.8) | 0.973 |
| BMI, kg/m2 | 27.6 (23.6-33.0) | 27.5 (23.5-32.9) | 0.033 |
| APACHE IV score | 53.0 (40.0-70.0) | 54.0 (40.0-72.0) | <0.001 |
| Past history of VTE, n (%) | 3685 (5.1) | 1413 (3.9) | <0.001 |
| History of cancer, n (%) | 10,809 (14.9) | 5259 (14.5) | 0.102 |
| Glasgow coma scale | 14.0 (10.0-15.0) | 14.0 (10.0-15.0) | <0.001 |
| Principal diagnosis on admission, n (%) |  |  |  |
| Cardiovascular condition | 19,353 (26.6) | 10,358 (28.5) | <0.001 |
| Respiratory condition | 11,982 (16.5) | 5923 (16.3) | 0.512 |
| Gastrointestinal condition | 7470 (10.3) | 3718 (10.2) | 0.889 |
| Renal condition | 1295 (1.8) | 574 (1.6) | 0.017 |
| Neurologic condition | 13,238 (18.2) | 6402 (17.6) | 0.023 |
| Sepsis | 10,145 (13.9) | 5069 (14.0) | 0.940 |
| Metabolic condition | 2981 (4.1) | 1415 (3.9) | 0.113 |
| Trauma | 3528 (4.9) | 1417 (3.9) | <0.001 |
| Other condition | 2750 (3.8) | 1426 (3.9) | 0.231 |
| Disease of patient, n (%) |  |  |  |
| Cancer | 3169 (4.4) | 1843 (5.1) | <0.001 |
| Respiratory failure | 17,244 (23.7) | 9625 (26.5) | <0.001 |
| Heart failure | 6776 (9.3) | 3535 (9.7) | 0.025 |
| End stage renal disease | 2096 (2.9) | 1078 (3.0) | 0.415 |
| Sepsis | 10,290 (14.1) | 5697 (16.0) | <0.001 |
| Treatments, n (%) |  |  |  |
| Mechanical ventilation | 26,963 (37.1) | 14,412 (39.7) | <0.001 |
| Central venous catheter | 10,004 (13.8) | 5385 (14.8) | <0.001 |
| Vasopressor | 12,848 (17.7) | 8210 (22.6) | <0.001 |
| Sedative | 25,915 (35.6) | 14,786 (40.7) | <0.001 |
| Transfusion of blood product |  |  |  |
| Platelet | 1460 (2.0) | 883 (2.4) | <0.001 |
| Fresh frozen plasma | 2601 (3.6) | 1436 (4.0) | 0.002 |
| Packed red blood cells | 6863 (9.4) | 3586 (9.9) | 0.019 |
| Laboratory test results |  |  |  |
| Hematocrit, % | 37.5 (32.7-42.0) | 37.1 (32.2-41.6) | <0.001 |
| Hemoglobin, g/dl | 12.4 (10.6-14.0) | 12.3 (10.5-13.9) | <0.001 |
| Platelet, K/uL | 221.0 (169.0-285.0) | 221.0 (169.0-287.0) | 0.901 |
| While blood cells, K/uL | 12.3 (9.0-17.0) | 12.1 (8.7-16.8) | <0.001 |
| Albumin, g/dL | 3.4 (2.9-3.8) | 3.3 (2.8-3.7) | <0.001 |
| Blood urea nitrogen, mg/dL | 22.0 (15.0-36.0) | 22.0 (15.0-37.0) | <0.001 |
| Creatinine, mg/dL | 1.1 (0.8-1.8) | 1.1 (0.8-1.8) | <0.001 |
| INR | 1.4 (1.1-1.9) | 1.4 (1.1-1.8) | <0.001 |
| PT, s | 15.9 (13.5-21.4) | 15.8 (13.4-21.3) | <0.001 |
| PTT, s | 35.2 (29.4-42.6) | 35.1 (29.3-42.4) | <0.001 |
| Total bilirubin, mg/dL | 0.6 (0.4-1.0) | 0.7 (0.4-1.1) | <0.001 |
| ALT, U/L | 26.0 (16.0-45.0) | 27.0 (18.0-47.0) | <0.001 |
| AST, U/L | 30.0 (20.0-58.0) | 30.0 (20.0-60.0) | 0.563 |
| VTE prophylaxis method, n (%) |  |  |  |
| Pharmacologic prophylaxis | 36,964 (50.8) | 18,699 (51.5) | 0.031 |
| Graduated compression stockings | 33,613 (46.2) | 17,263 (47.6) | <0.001 |

Abbreviations: VTE, venous thromboembolism; APACHE IV score, Acute Physiology and Chronic Health Evaluation IV score; INR, international standard ratio; PT, prothrombin time; PTT, partial thromboplastin time; ALT, alanine aminotransferase; AST, aspartate transaminase.

**Figure S1: Percentage of missing variables.**


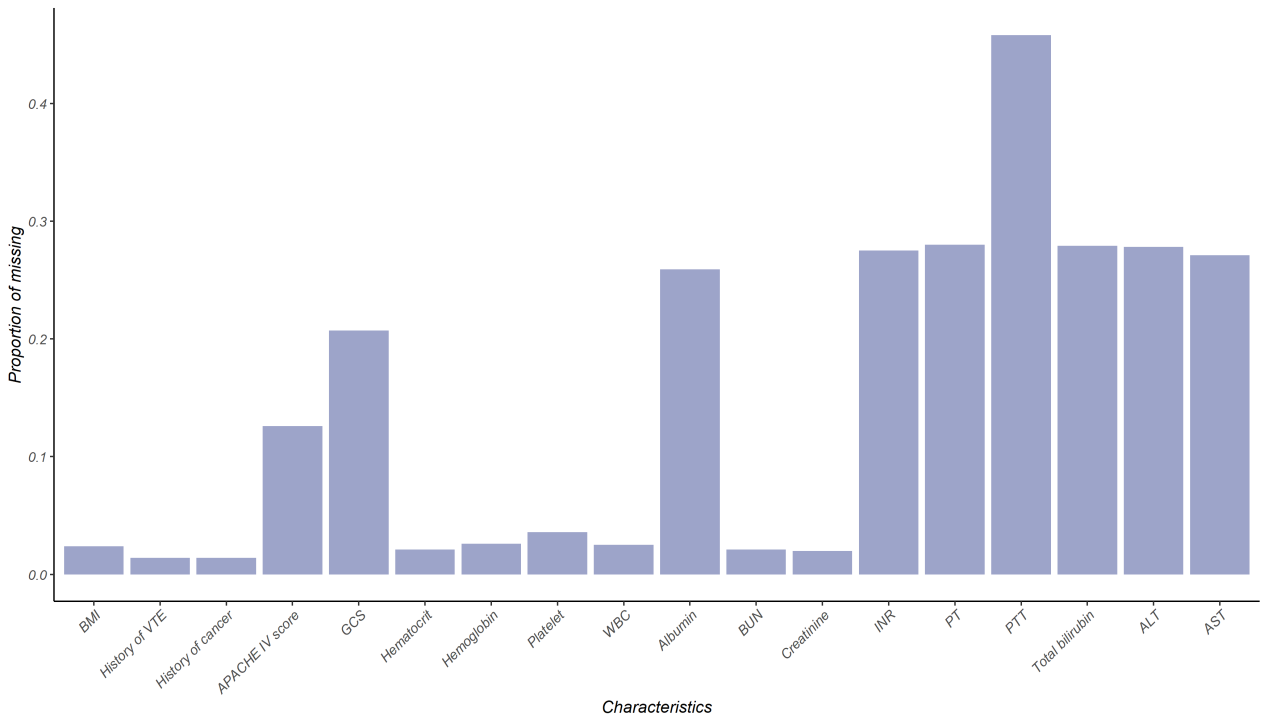


In sFigure 1, we summarized the percentage of missing data. The missing percentages for each variable were as follows: BMI (2.4%), History of VTE (1.4%)、History of cancer (1.4%), APACHE IV score (12.6%), GCS (25.7%), Hematocrit (2.1%), Hemoglobin (2.6%), Platelet (3.6%), WBC (2.5%), Albumin (25.9%), BUN (2.1%), Creatinine (2%), INR (27.5%), PT (28%), PTT (45.8%), Bilirubin (27.9%), ALT (27.8%), AST (27.1%). Abbreviations: BMI, body mass index; VTE, venous thromboembolism; APACHE IV score, Acute Physiology and Chronic Health Evaluation IV score; GCS, glasgow coma scale; WBC, white blood cell; Bun, blood urea nitrogen; INR, international standard ratio; PT, prothrombin time; PTT, partial thromboplastin time; ALT, alanine aminotransferase; AST, aspartate transaminase.

**Figure S2: Relationship between age, albumin, hematocrit, hemoglobin and predicted outcomes.**


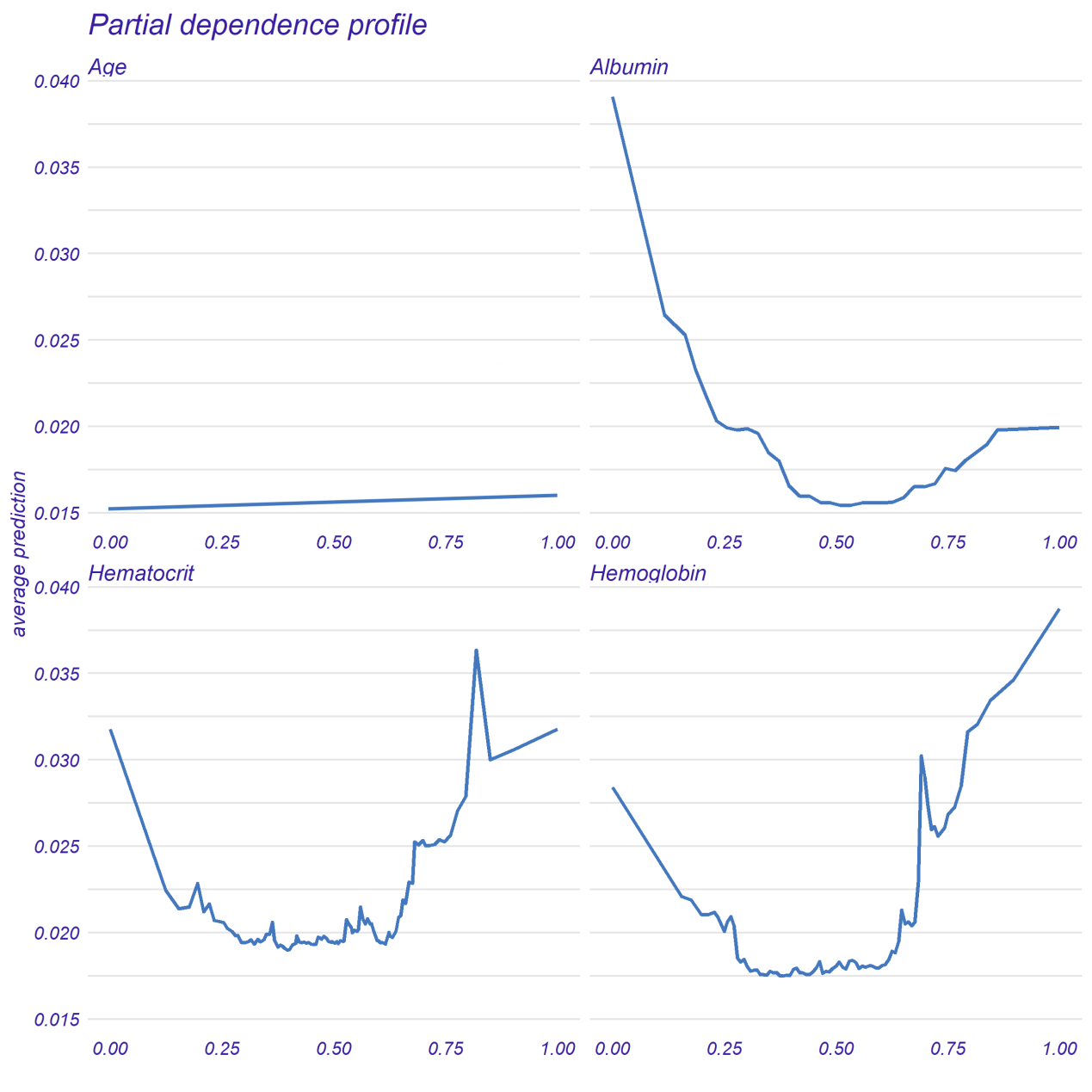


This figure was output by the DALEX package. It showed the relationship between age, albumin, hematocrit, hemoglobin and predicted outcomes. Higher age was associated with an increased risk of VTE. And albumin, hematocrit, and hemoglobin were associated with an increased risk of VTE in a U-shaped curve.

**Figure S3: Relationship between BMI, INR, platelet, PT, PTT, WBC and predicted outcomes.**


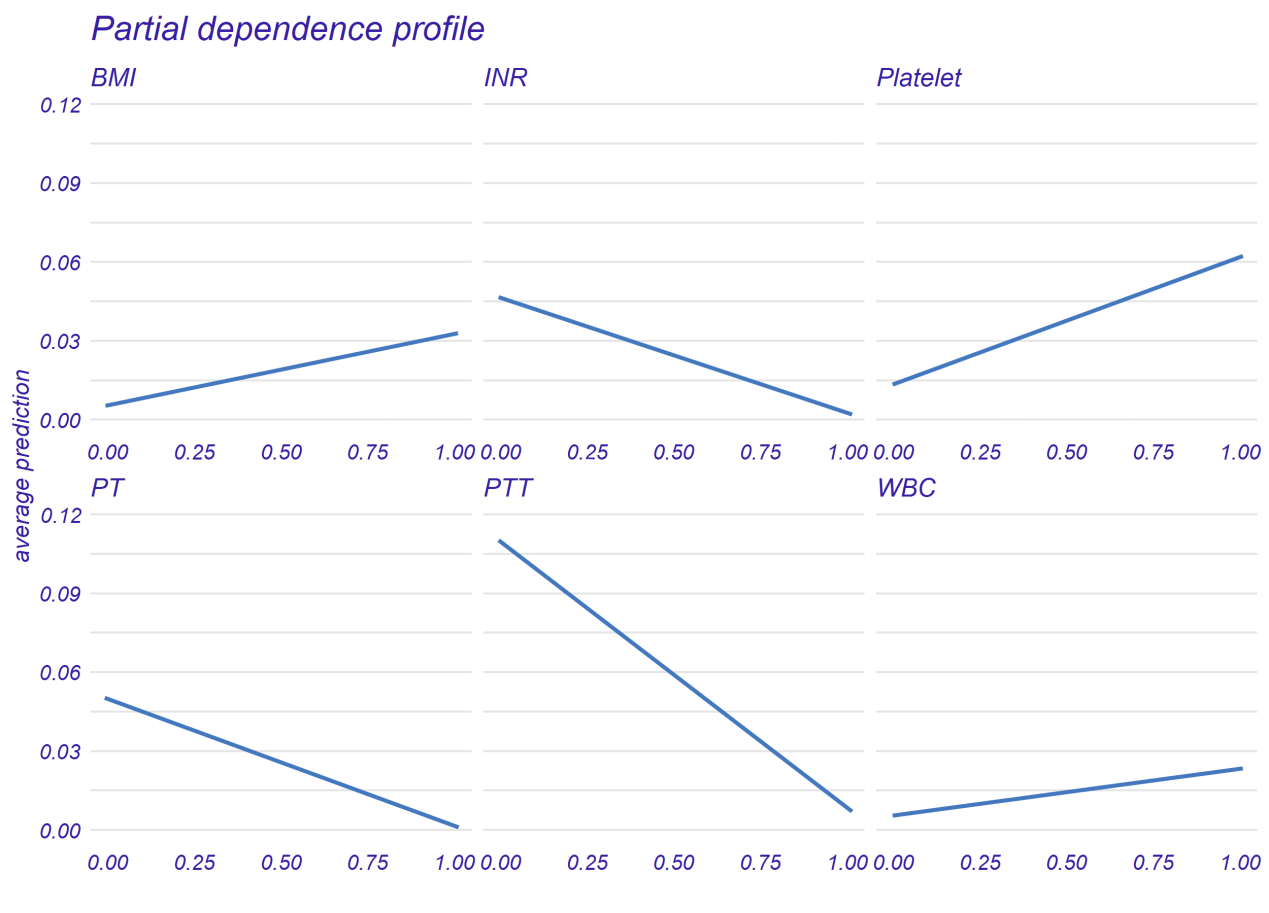


This figure was output by the DALEX package. It showed the relationship between BMI, INR, platelet, PT, PTT, WBC and predicted outcomes. Higher BMI, platelet count and white blood cell count were associated with an increased risk of VTE. Lower INR, PT and PTT were associated with an increased risk of VTE. Abbreviations: BMI, body mass index; INR, international standard ratio; PT, prothrombin time; PTT, partial thromboplastin time; WBC, while blood cells.

**Figure S4: Relationship between ALT, AST, creatinine, total bilirubin and predicted outcomes.**


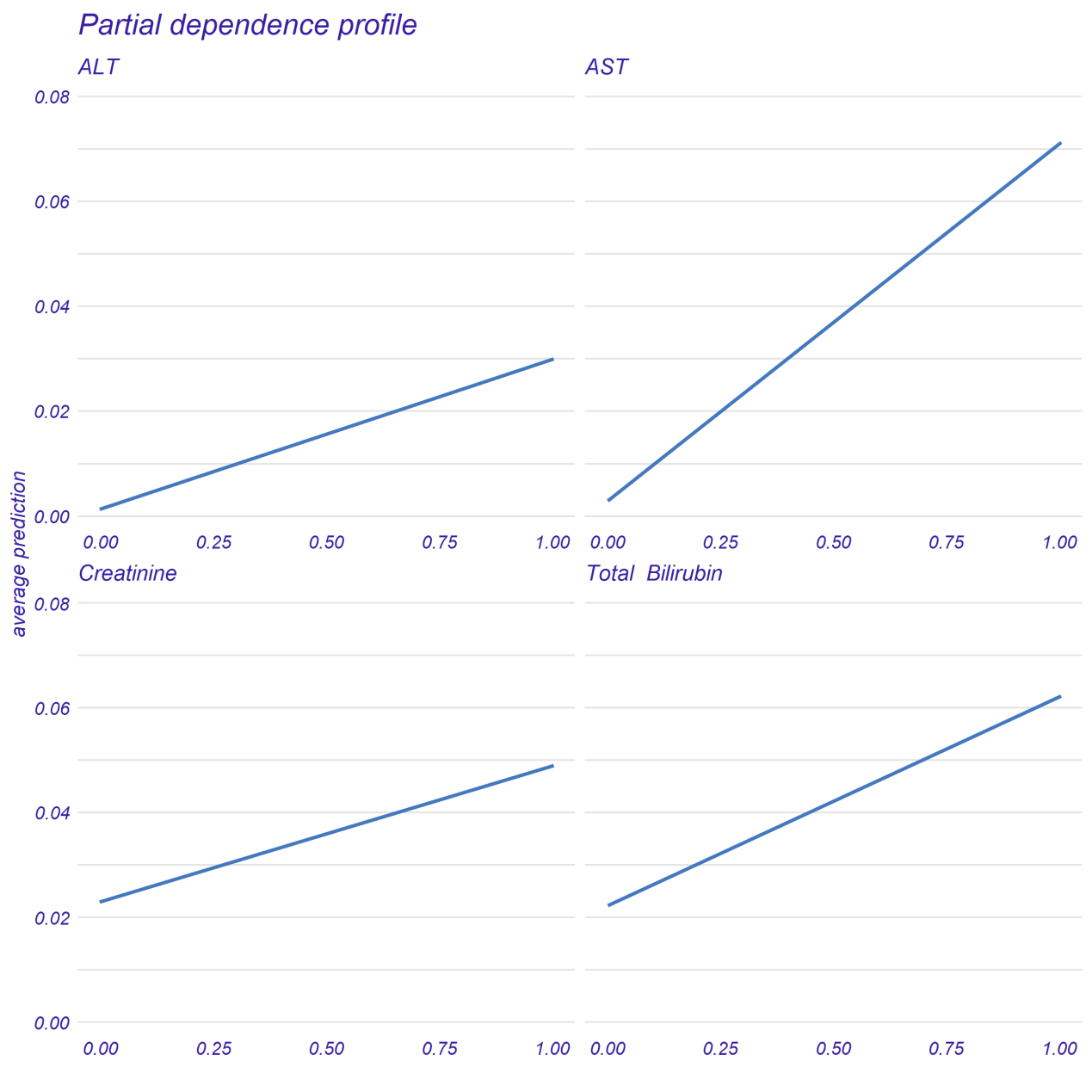


This figure was output by the DALEX package. It showed the relationship between ALT, AST, serum creatinine, total bilirubin and predicted outcomes. The risk of VTE increased with elevated ALT, AST, serum creatinine, and total bilirubin. Abbreviations: ALT, alanine aminotransferase; AST, aspartate transaminase.

**Figure S5: Relationship between cancer, cancer history, gender, heart failure, respiratory failture, vte history and predicted outcomes.**


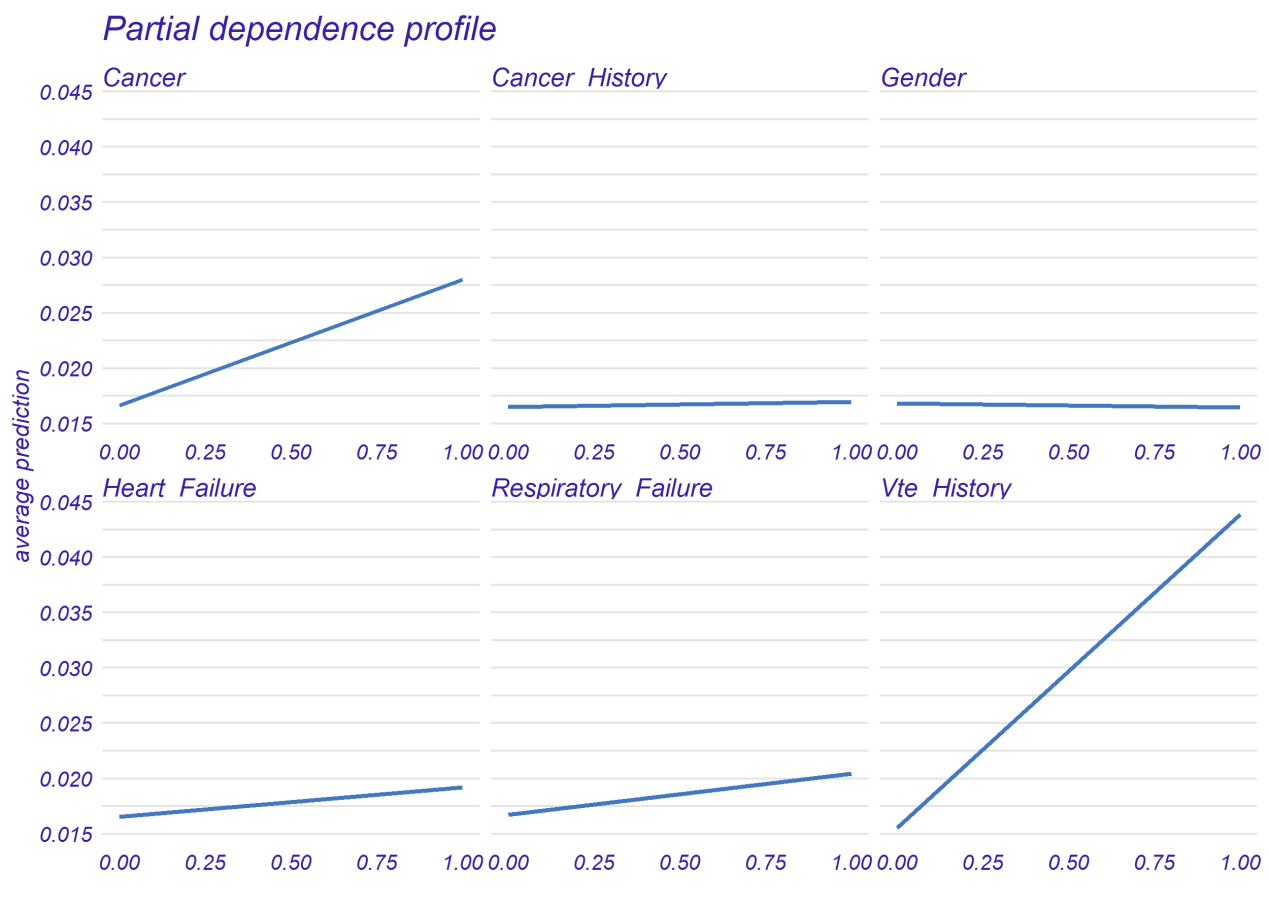


This figure was output by the DALEX package. It showed the relationship between cancer, cancer history, gender, heart failure, respiratory failure, VTE history and predicted outcomes. A history of prior VTE, a diagnosis of cancers, heart failure, respiratory failure were helpful in predicting VTE. Gender and cancer history were not strongly associated with VTE prediction. Abbreviations: VTE, venous thromboembolism.

**Figure S6: Relationship between CVC, RBW input, sepsis, mechanical ventilation and predicted outcomes.**


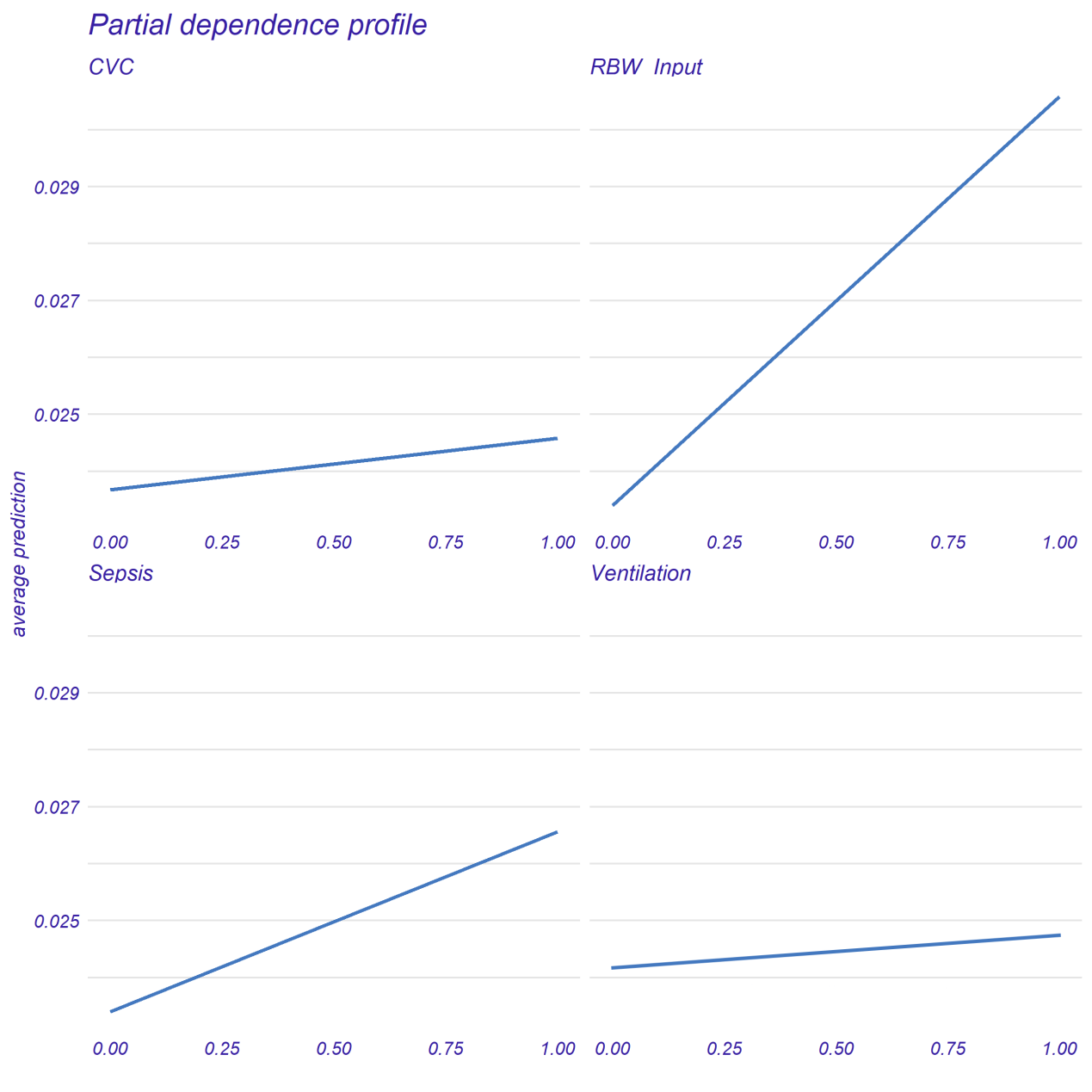


This figure was output by the DALEX package. It showed the relationship between CVC, RBW input, sepsis, mechanical ventilation and predicted outcomes. A diagnosis of sepsis, treatment with CVC, mechanical ventilation, and transfusion of packed red blood cells were helpful in predicting VTE. Abbreviations: CVC, central venous catheter; RBW, packed red blood cells.

**Figure S7: Explaining of patient prediction results.**

**
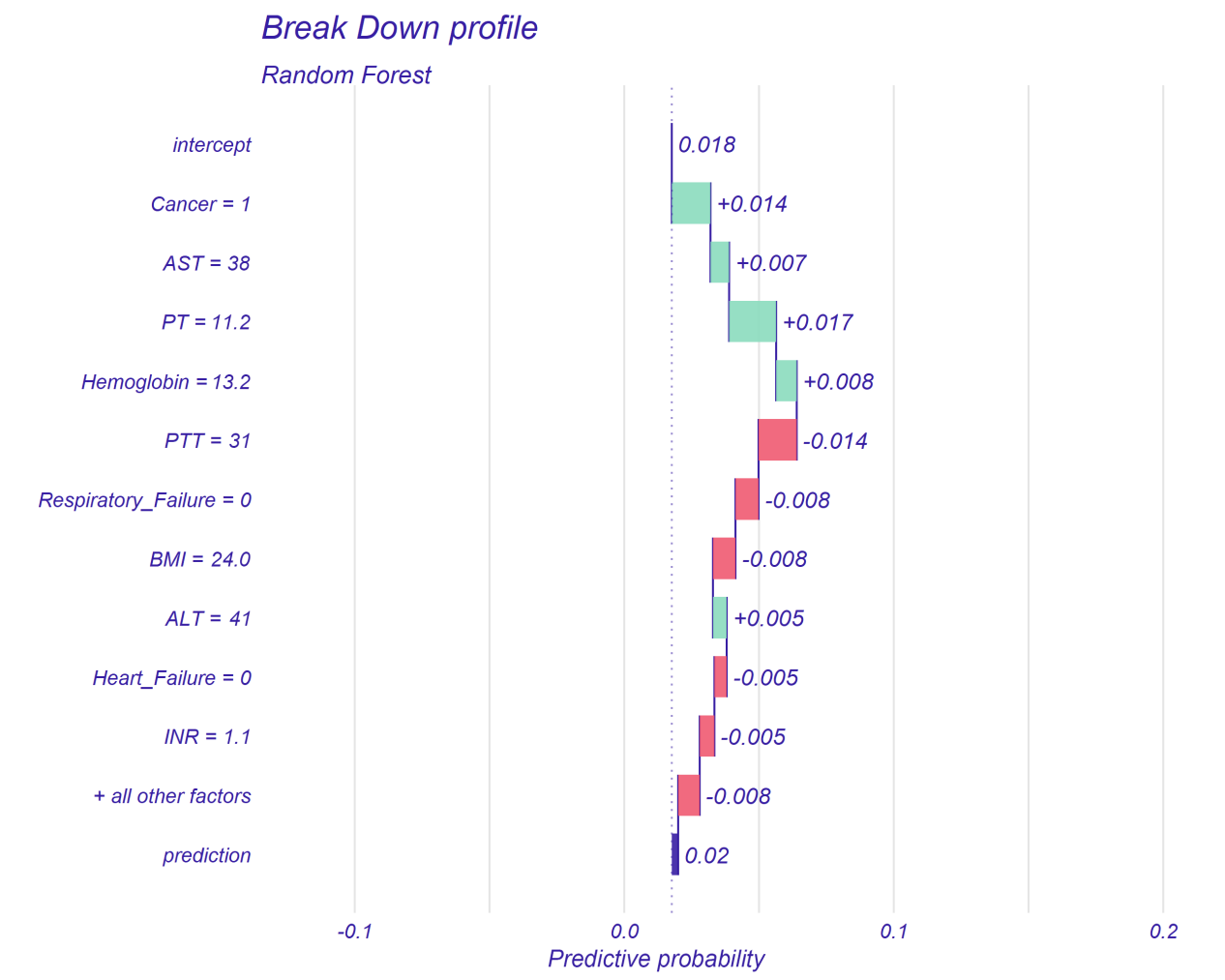
**

This figure was made with the DALEX package for explaining random forest model predictions. This patient was a 72-year-old male with a BMI of 24.0. He had no previous history of VTE but did have a history of cancer, with a diagnosis of cancer and sepsis. Laboratory results on the first day of ICU admission showed a hematocrit of 42.1%, hemoglobin level of 13.2 g/dL, platelet count of 196 K/uL, white blood cell count of 9.8 K/uL, and albumin level of 2.8 g/dL. His serum creatinine was 0.6 mg/dL, INR was 1.1, PT was 11.2 s, PTT was 31 s, total bilirubin was 0.6 mg/dL, ALT was 41 U/L, and AST was 38 U/L. He did not require mechanical ventilation and was not transfused with packed red blood cells or CVC. Patient PT and comorbid cancers increased the risk of VTE, but PTT, uncomplicated respiratory failure, and BMI offset the risk. The final model predicted a 2% incidence of VTE. The ML model predicted that the patient would not develop VTE; the result was that the patient did not develop VTE while in the ICU (true negative). Abbreviations: AST, aspartate transaminase; PT, prothrombin time; PTT, partial thromboplastin time; BMI, body mass index; ALT, alanine aminotransferase; INR, international standard ratio.

**Figure S8: Explaining of patient prediction results.**


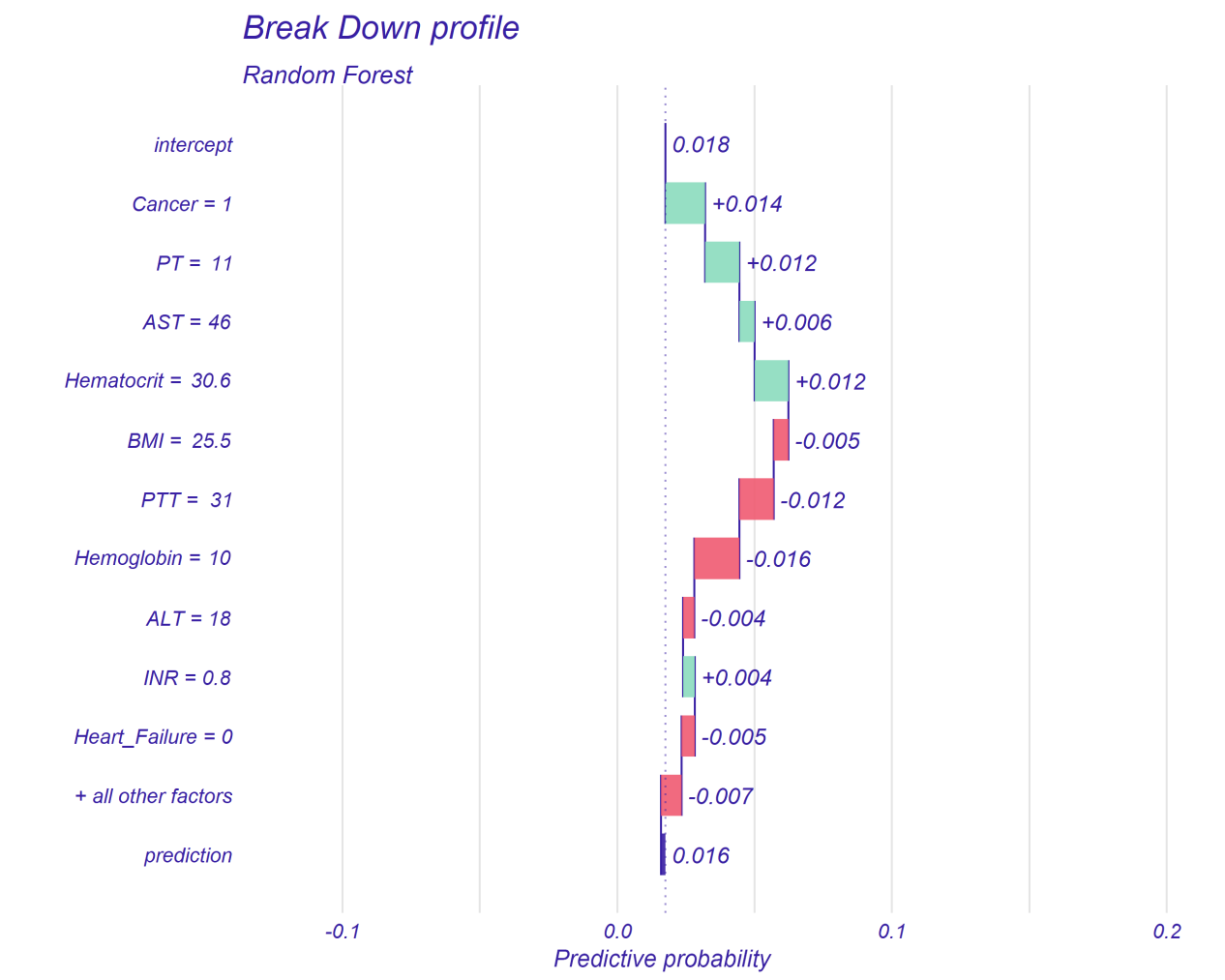


This figure was made with the DALEX package for explaining random forest model predictions. This patient was a 56-year-old female with a BMI of 25.5. She had no previous history of VTE but did have a history of cancer, with a diagnosis of cancer. Laboratory indices on the first day of ICU admission revealed a hematocrit of 30.6%, hemoglobin level of 10 g/dL, platelet count of 192 K/uL, white blood cell count of 7.8 K/uL, and albumin level of 3.7 g/dL. Her serum creatinine level was 0.9 mg/dL, INR was 0.8, PT was 11 s, PTT was 31 s, total bilirubin was 0.3 mg/dL, ALT was 18 U/L, and AST was 46 U/L. She did not require mechanical ventilation treatment and was not transfused with packed red blood cells or CVC inserted. The model predicted a 1.6% incidence of VTE in this patient. Comorbid cancers, PT, and hematocrit increased the predicted probability of VTE, but hemoglobin, PTT, and BMI decreased the risk of VTE. The ML model predicted that the patient would not develop VTE; the result was that the patient did not develop VTE while in the ICU (true negative). Abbreviations: PT, prothrombin time; AST, aspartate transaminase; BMI, body mass index; PTT, partial thromboplastin time; ALT, alanine aminotransferase; INR, international standard ratio.

**Figure S9: Explaining of patient prediction results.**


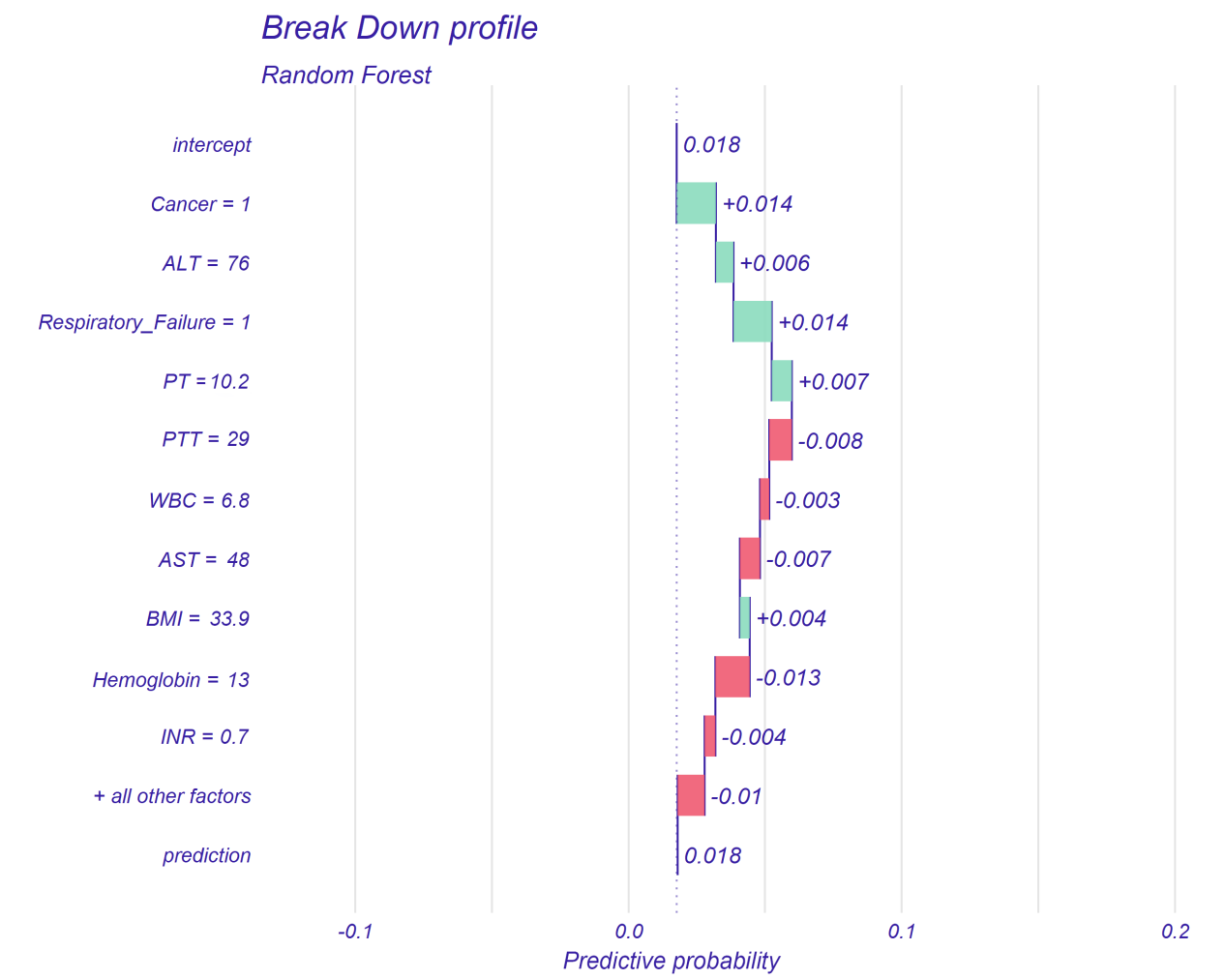


This figure was made with the DALEX package for explaining random forest model predictions. This patient, a 52-year-old male with a BMI of 33.9, had no previous history of VTE but did have a history of cancer along with diagnoses of cancer, respiratory failure, and sepsis. Laboratory markers on the first day of ICU admission showed a hematocrit of 40.8%, hemoglobin level of 13 g/dL, platelet count of 383 K/uL, leukocyte count of 6.8 K/uL, and albumin level of 3.1 g/dL. His serum creatinine was 0.9 mg/dL, INR was 0.7, PT was 10.2 s, PTT was 29 s, total bilirubin was 0.5 mg/dL, ALT was 76 U/L, and AST was 48 U/L. He received mechanical ventilation treatment and did not require a transfusion of packed red blood cells or CVC inserted. The ML model predicted a 1.8% incidence of VTE in this patient, with the predicted outcome being that the patient did not develop VTE and the actual outcome being that the patient developed VTE while in the ICU (false negative). This patient's comorbidities, such as cancer and respiratory failure, increased the risk of VTE, but hemoglobin, PTT, and AST offset the risk of VTE. Abbreviations: ALT, alanine aminotransferase; PT, prothrombin time; PTT, partial thromboplastin time; WBC, while blood cells; AST, aspartate transaminase; BMI, body mass index; INR, international standard ratio.
